# Periodontal Disease and Risk of Heart Failure: A Systematic Review and Meta‐Analysis

**DOI:** 10.1155/ijod/3288710

**Published:** 2026-04-22

**Authors:** German Valenzuela-Rodríguez, Carlos Diaz-Arocutipa, Frank Mayta-Tovalino, Adrian V. Hernandez

**Affiliations:** ^1^ Internal Medicine and Cardiology Services, Clinica Delgado, Lima, Peru; ^2^ Unidad de Revisiones Sistematicas y Meta-Analisis (URSIGET), Vicerrectorado de Investigacion, Universidad San Ignacio de Loyola, Lima, Peru, usil.edu.pe; ^3^ Health Outcomes, Policy, and Evidence Synthesis (HOPES) Group, University of Connecticut School of Pharmacy, Storrs, Connecticut, USA, uconn.edu

**Keywords:** heart failure, meta-analysis, periodontal disease, systematic review

## Abstract

Periodontal disease (PD) has been hypothesized to influence the risk of heart failure (HF), but the evidence remains inconclusive. We conducted a systematic review to evaluate the association between PD and HF. PubMed, Embase, Scopus, and Web of Science were searched from inception to October 2024. We selected cohort studies assessing the association between PD and HF in adults. Random effects models were used for all meta‐analyses, and relative risks (RRs) were used to describe such associations. The risk of bias was evaluated using the Newcastle–Ottawa scale (NOS). Seven cohort studies (*n* = 376,166), including five prospective and two retrospective studies, were included. The studies had a mean age range of 43–65.3 years, with male representation ranging from 31.7% to 78%, and follow‐up durations of 5.4–13 years. Among these, four studies were classified as low risk of bias, and three with unclear risk of bias. PD was associated with an increased risk of HF (crude RR 1.58, 95% confidence interval [CI] 1.20–2.07, *p*  < 0.01). Our findings suggest a potential association between PD and HF risk similar to a previous meta‐analysis with fewer included individuals and without a quality assessment of the cohort studies. However, the limited number of cohort studies and significant heterogeneity warrant cautious interpretation. Further high‐quality, longitudinal studies are needed to elucidate the nature and mechanisms of this relationship.

## 1. Introduction

Periodontitis is a chronic infectious and inflammatory disease characterized by the proliferation of pathogenic bacteria, often leading to systemic dissemination and the release of pro‐inflammatory cytokines into the bloodstream, with evidence of elevated levels of interleukin (IL)‐6, IL‐1β, tumor necrosis factor (TNF)‐α, and C‐reactive protein [1]. Periodontitis has been associated with several cardiovascular diseases, including subclinical cardiovascular disease, myocardial infarction, stroke, peripheral artery disease, atrial fibrillation, and heart failure (HF) [1].

HF is a clinical syndrome with diverse etiologies and a complex pathophysiology, with symptoms and/or signs caused by structural and/or functional cardiac abnormality and supported by elevated natriuretic peptides and/or objective evidence of pulmonary or systemic congestion [2].

The prevalence of HF is approximately 1%–2% in the general population and rises to more than 10% among individuals over 70 years of age [3]. The incidence varies among regions and countries. Hospitalization due to HF represents 1%–2% of all hospital admissions in the Western world and is the most frequent cause of hospitalization among individuals over 65 years of age [4]. Emerging evidence suggests that systemic inflammation, such as that observed in periodontitis, may play a critical role in the pathogenesis of HF. Notably, elevated levels of *Porphyromonas gingivalis*—a key periodontal pathogen—have been detected in individuals with HF, suggesting an association of periodontitis in the inflammatory cascade associated with HF [5]. Inflammation contributes to HF through multiple pathways, including its impact on pathogenesis; underlying comorbidities such as diabetes and obesity; pathological substrates such as endothelial dysfunction and atherosclerosis; and disease progression and outcomes [6].

Epidemiological studies and a previous meta‐analysis including only three cohort studies have reported a higher prevalence of severe periodontitis among individuals with HF compared to the general population, suggesting a potential link between these conditions and underscoring the need for further investigation to quantify the degree of association [7]. Therefore, our aim was to assess the association between periodontitis and HF by analyzing the best available data published in the most recent cohort studies.

## 2. Methods

### 2.1. Study Population

This systematic review was reported following the 2020 Preferred Reporting Items for Systematic Reviews and Meta‐Analyses (PRISMA) statement [8]. The protocol was registered in the PROSPERO registry (CRD42024605010).

We searched the electronic databases PubMed, Embase, Scopus, and Web of Science until October 2024 to identify relevant studies reporting the association between periodontal disease (PD) and HF. Detailed search strategies are available in the Supporting Information section. In addition, reference lists of all retrieved articles were manually reviewed to identify additional eligible studies.

#### 2.1.1. Inclusion and Exclusion Criteria

Two independent reviewers (German Valenzuela‐Rodríguez and Carlos Diaz‐Arocutipa) screened titles, abstracts, and full texts of retrieved studies. Disagreements were resolved through discussion or adjudication by a third reviewer (Frank Mayta‐Tovalino). Studies were considered potentially eligible if they were cohort or case–control studies that reported the association between PD or periodontitis and HF, with no exclusion based on the follow‐up period. Case reports or case series were excluded. Patients with other cardiovascular or metabolic comorbidities were not excluded.

### 2.2. Study Execution Method

The primary outcome was HF, as defined by each cohort study. Definitions of PD or periodontitis and HF varied across studies and are described in Table 1. International Classification of Diseases (ICD) codes for PD were defined only in two studies [11, 15], and definitions in other studies often relied on questionnaires, medical records, and/or electronic databases.

**Table 1:** Definitions of PD or periodontitis and HF.

Author, year (reference)	Definition of PD or periodontitis	Definition of HF
Norhammar et al., 2024 [9]	Periodontitis: dental examination including a panoramid dental radiograph according to a standardized protocol	Hospitalization for heart failure (non‐specified code)
Huh et al., 2023 [10]	Periodontal diseases: results of an oral examination status	Code I50
Nabila et al., 2023 [11]	Periodontal diseases: obtained by Health Insurance Review and Assessment Service (HIRA) with two visits in a month (diagnostic code K05 or health‐claim‐of‐treatment codes: U2240, U1010, U1020, U1051,U1052, U1071, U1072, U1081, U1082, U4412, U4413, U4414)	Hospitalization for at least 2 days because of heart failure, Code I50
Molinsky et al., 2022 [12]	Periodontal diseases: by periodontal examinations measuring probing depth, gingival recession and bleeding at six sites per tooths following periodontal profile class (PPC)	HF‐related ICD‐9 discharged code
Walther et al., 2022 [13]	Periodontitis: using a comprehensive oral examination performed by certified study nurses supervised by dentists, graded by Eke and Page scale	Based on 2021 guidelines: clinical self‐reported history of HF, HF medication, laboratory data or echocardiographic criteria
Yan et al., 2022 [14]	Periodontitis: using the extent and severity of probing depth (PD) and attachment loss	Physician definition (yes or no)
Chang et al., 2020 [15]	Periodontal diseases: using these codes: acute periodontitis (K052), chronic periodontitis (K053), peridontosis (K054), other periodontal disease (K055), unspecified periodontal disease (K056), diagnosed by dentists and when more than two claims ocurred	Code I50 with at least two claims per year

#### 2.2.1. Data Extraction

Two reviewers (German Valenzuela‐Rodríguez and Carlos Diaz‐Arocutipa) independently extracted data from the included studies, and discrepancies were resolved by discussion between reviewers. The following information was obtained from each study: author, year of publication, countries, number of patients studied, study design, patient characteristics (mean age, percentage of male participants, smoking, arterial hypertension, and diabetes), number of individuals with/without PD and HF, and duration of follow‐up.

#### 2.2.2. Study Quality Assessment

We used the NOS to assess the risk of bias in case–control and cohort studies. The NOS tool consists of three domains: selection, comparability, and outcome assessment. It assigns a maximum of four points for selection, two for comparability, and three for outcome assessment [16]. NOS scores >7 were considered to indicate low risk of bias, scores of 4–6 were regarded as unclear risk of bias, and scores of <3 points as high risk of bias. A joint reevaluation of the original articles addressed any discrepancies [17].

### 2.3. Data Analysis Method

All meta‐analyses were conducted using random effects models with the inverse‐variance method [18]. The association between PD and HF was reported as relative risks (RRs) with their 95% confidence intervals (CIs). Our primary analysis was based on all studies fulfilling the inclusion and exclusion criteria. We primarily planned meta‐analyzing adjusted effects of the association between PD and HF if those effects came from studies with little methodological heterogeneity and were adjusted for a similar set of confounders. If those conditions were not fulfilled, then we analyzed crude effects and described qualitatively adjusted effects and confounders across studies. Two sensitivity analyses were conducted: (i) in subset of studies with more similar populations and outcomes and (ii) by excluding studies that used periodontitis instead of PD. Statistical heterogeneity was evaluated using the *I* ^2^ statistic and was defined as follows: *I* ^2^ >30% low, 30%–60% moderate, and >60% high heterogeneity [19]. We used the meta package in R version 4.1.2 (www.r-project.org) for all analyses.

## 3. Results

**Figure 1:**
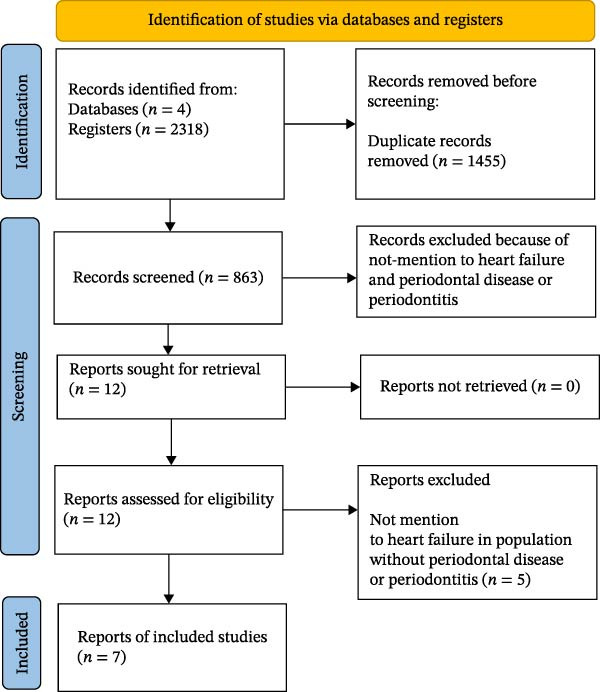
Flowchart of study selection.

A total of 2318 records were screened during the initial assessment. After excluding 2311 records, 12 studies were identified for full‐text review (Figure 1). Of these, five studies were excluded because they did not provide data on HF in individuals without PD or periodontitis. Consequently, seven cohort studies met the inclusion criteria and were included in the primary analysis [9–12, 17–19]. These comprised five prospective and two retrospective studies.

Three studies were excluded from our secondary analysis due to methodological considerations. Norhammar et al. [9] examined a cohort of patients with a first myocardial infarction recruited across 17 Swedish hospitals over several years. Huh et al. [10] analyzed patients with type 2 diabetes in a nationwide Korean cohort with follow‐up from 2008 to 2017. Finally, Nabila et al. [11] used data from the Korean National Health and Nutrition Examination Survey (2007–2015) but combined coronary heart disease and HF into a single outcome, limiting its applicability to our specific research question [9–12, 17–19].

The seven included cohort studies were published between 2020 and 2024 (Table 2). Studies provided data from 376,166 patients, with a mean age ranging from 43 to 65.3 years. The proportion of male participants varied between 31.7% and 78%, and there was heterogeneity in the prevalence of cardiovascular risk factors like smoking, arterial hypertension, and diabetes mellitus. Follow‐up durations ranged from 5.4 to 13 years. Two studies were conducted in Europe (Sweden and Germany), four in Asia (Korea and China), and one in the United States. Definitions of periodontitis and PD across included studies are described in detail in Table 1. Adjusted effect measures of the association between PD/periodontitis and HF are shown in Table 3. All studies reported adjusted effects, including different sets of covariates and using different statistical methods.

**Table 2:** Characteristics of the included cohort studies.

Author, year (reference)	Year	Country	Sample size	Age	Male gender (%)	Smoking (%)	Arterial hypertension (%)	Diabetes mellitus (%)	Type of cohort study	Follow‐up in years
Norhammar et al. [9]	2024	Sweden	1587	65+	78	73	39	12	Prospective	9.9
Huh et al. [10]	2023	Korea	173,927	65.3+	74.2	28.8	57.6	50.4	Prospective	11
Nabila et al. [11]	2023	Korea	14,315	NA	31.7	33.7	4.97	6.7	Prospective	5.4
Molinsky et al. [12]	2022	USA	6707	63+	51	59	38	14	Prospective	13
Walther et al. [13]	2022	Germany	6209	64+	52.1	18.9	64.6	7.6	Prospective	10
Yan et al. [14]	2022	China	13,202	43+	60.9	63.8	65.4	17.4	Retrospective	NA
Chang et al. [15]	2020	Korea	161,286	52.2+	61.2	25.1	38.9	9	Retrospective	10.5

*Note:* ^+^, mean; ^++^, median.

Abbreviation: NA, not available.

**Table 3:** Adjusted effect measures of the association between PD and HF.

Author, year (reference)	Adjusted effect estimate (95% CI)	Model to calculate adjusted effect	Adjusted variables
Norhammar et al., 2024 [9]	aHR: 1.26 (1.01–1.57) [periodontal disease]	Cox regression analysis	Age, smoking, and diabetes
Huh et al., 2023 [10]	aHR 1.17 (1.08–1.26) (periodontal disease, Model 1)	Cox proportional hazard regression analysis	Age, sex, smoking, alcohol consumption, physical activity, income, body mass index, hypertension, dyslipidemia, chronic kidney disease, cardiovascular disease, and antidiabetic medications
Nabila et al., 2023 [11]	aHR 1.27 (1.03–1.56) (periodontal disease and chronic disease, Model 1)	Cox proportional hazard regression analysis	Sex, income level, age, residential area, smoking status, smoking pack‐years, alcohol consumption, and physical activity, BMO biomarkers, dental behavior, oral health at baseline, history of other diseases.
Molinsky et al., 2022 [12]	IR 1.79 (1.41–2.27) (model 1, combined HFpEF or HFrEF)	Cox proportional hazard regression analysis	Age, sex, race/center, education, insurance model, cigarette status, physical activity, body mass index, LDL cholesterol, hypertension medication, CHD, diabetes, systolic BP, change in CRP, baseline in NT‐proBNP
Walther et al., 2022 [13]	OR 1.64 (0.88–3.06) (severe periodontitis)	Unspecified multivariable regression models	Age, female sex, body mass index, smoking, diabetes, hypertension, atrial fibrillation and coronary artery disease
Yan et al., 2022 [14]	OR 5.72 (3.76–8.72) (Model 1, unadjusted)	Weighted logistic regression	Gender, age, race, body mass index, poverty income ratio, education, marital status, smoking status, drinking status, hypertension, diabetes, stroke and asthma
Chang et al., 2020 [15]	aHR 1.03 (0.96–1.10) (multivariable adjusted model 3)	Unspecified multivariable regression models	Age, sex, socioeconomic status, regular exercise, alcohol consumption, body mass index, hypertension, diabetes mellitus, dyslipidemia, smoking status, renal disease, history of cancer, systolic blood pressure, fasting blood sugar, liver panel, proteinuria, periodontal disease, tooth brushing, dental visit, professional dental cleaning, number of missing teeth

Abbreviations: aHR, adjusted hazard ratio; BP, blood pressure; CHD, coronary heart disease; CRP, C‐reactive protein; HFpEF, heart failure with preserved ejection fraction; HFrEF, heart failure with reduced ejectiin fraction; IR, incidence rate; OR, odds ratio.

Four studies were classified as low risk of bias, and three were classified as unclear risk of bias according to the NOS tool (Table 4). Sources of bias were mostly in the selection domain (demonstration that the outcome of interest was not present at baseline) and in the outcome section (assessment of outcome, incomplete follow‐up, and adequacy of follow‐up cohorts).

**Table 4:** Quality assessment of risk of bias across included studies with the Newcastle–Ottawa scale.

Author, year (reference)	Selection				Comparability		Outcome			Total
	Representativeness of exposed cohort	Selection of nonexposed cohort	Ascertainment of exposure	Demonstration that outcome of interest was not present	Comparability of cohorts	Study controls for any additional factors	Assessment of outcome	Was follow‐up long enough for outcomes	Adequacy of follow‐up of cohorts	
Norhammar et al., 2024 [9]	1	1	1	0	1	1	0	1	1	7
Huh et al., 2023 [10]	1	1	1	0	1	1	0	1	1	7
Nabila et al., 2023 [11]	1	1	1	0	1	1	0	0	1	6
Molinsky et al., 2022 [12]	1	1	1	0	1	1	1	1	1	8
Walther et al., 2022 [13]	1	1	1	0	1	1	1	0	0	6
Yan et al., 2022 [14]	1	1	0	0	1	1	1	0	0	5
Chang et al., 2020 [15]	1	1	1	0	1	1	1	1	1	8

### 3.1. Meta‐Analysis Results

**Figure 2:**
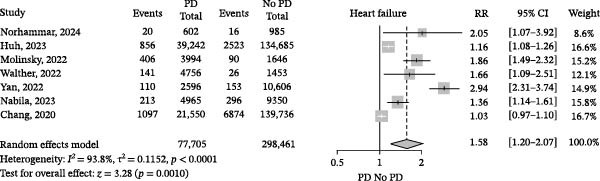
Association of periodontal disease (PD) and heart failure described with RR across all included studies. Squares represent the RR of each individual RCT, horizontal lines represent the 95% confidence intervals (CIs) of the RR, and diamonds are the RR of the overall random effects meta‐analysis.

**Figure 3:**
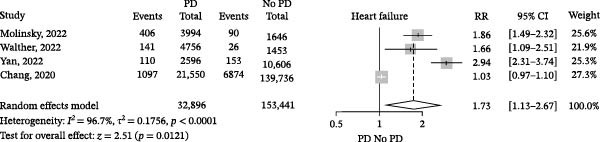
Association of periodontal disease (PD) and heart failure in four studies with similar populations and outcomes. Squares represent the RR of each individual RCT, horizontal lines represent the 95% confidence intervals (CIs) of the RR, and diamonds are the RR of the overall random effects meta‐analysis.

We pooled crude RRs because adjusted estimates differed substantially in terms of covariates and model specifications across studies, making them unsuitable for direct quantitative synthesis. PD/periodontitis was weakly associated with HF, with high statistical heterogeneity among studies (crude RR 1.58, 95% CI 1.20–2.07, *I* ^2^ = 93.8%, seven studies; Figure 2), suggesting that the strength of this association may vary substantially across clinical settings and populations. In contrast, when we included four studies with similar populations and outcomes, the crude RR was 1.73, and high statistical heterogeneity was also observed (95% CI 1.13–2.67, *I* ^2^ = 96,7%; Figure 3), indicating that even in more homogeneous cohorts, the clinical effect remains unstable and should be interpreted with caution. When excluding studies that described periodontitis instead of PD, we found that there was an association with HF (crude RR 1.30, 95% CI 1.02–1.66, *I* ^2^ = 90.6%, four studies; Figure S1). When analyzing studies assessing periodontitis, there was a higher association with HF (crude RR 2.26, 95% CI 1.58–3.23, *I* ^2^ = 65.9%, three studies, Figure S2).

## 4. Discussion

This meta‐analysis includes seven cohort studies (five prospective and two retrospective) obtained from four databases, with the largest number of patients included and applying a rigorous risk‐of‐bias assessment. Our results indicate a potential association between PD or periodontitis and an increased risk of HF. However, only three studies exhibited an unclear risk of bias but had significant heterogeneity in effect sizes and enrolled populations with varying demographic and clinical characteristics.

The role of the oral microbiome in systemic inflammatory diseases and cardiometabolic outcomes is increasingly recognized. Strong evidence links PD to atherosclerotic vascular disease, with proposed mechanisms including microbial dysbiosis, nitric oxide pathway disruption, and systemic inflammation [20].

The association between PD and HF has been less frequently studied. A longitudinal study with 13 years of follow‐up identified elevated levels of pro‐inflammatory markers such as TNF‐α and the presence of PD‐associated bacteria like *Treponema denticola* and *P. gingivalis* in individuals at increased cardiovascular risk [20, 21].

Additionally, older patients (>60 years of age) with treated periodontitis have demonstrated increased rates of major adverse cardiovascular events even after matching for other cardiovascular risk factors, supporting the possible dose‐dependent effect of periodontitis on MACE in this population [22]. Among patients with advanced HF (severe HF, heart transplantation, and left‐ventricular assist device), bacterial infections at the heart driveline and outside the heart, and sepsis were more prevalent during cardiovascular follow‐up of approximately 3 years, suggesting a potential interplay between PD‐related systemic inflammation and HF progression [23].

There is evidence that supports the coexistence of periodontitis and cardiovascular disease driven by shared risk factors such as obesity, type 2 diabetes, genetic factors, and environmental factors like smoking, oral hygiene, nutrition, and psychological stress, which could influence both conditions and may thus be potential confounders in the association between these diseases [24, 25].

Our study represents the second meta‐analysis to explore the association between PD or periodontitis and HF. A prior meta‐analysis that included one study with the definition of PD and two with the definition of periodontitis, reported as a letter to the editor, found an increased risk of HF similar to our findings (OR 1.58, 95% CI 1.15–2.18), with lower heterogeneity among the three selected studies published in 2022 (*I* ^2^ = 78.4%), but it was limited by a smaller study sample (13,039 individuals) and a lack of systematic assessment of study bias [26].

Some guidelines recommend early detection of periodontitis in primary care settings, considering the association between this condition and cardiovascular diseases, to promote its appropriate management and encourage healthy lifestyles [27].

Our study has some limitations. First, the number of studies specifically identifying HF as an endpoint remains limited; this situation makes main findings not conclusive. Second, population heterogeneity, including differences in age, comorbidity prevalence, and healthcare systems, as well as unclear outcome definitions, may confound the observed association between PD and HF; we performed sensitivity analysis by excluding three studies with these limitations and found that the significant higher risk of HF still was significant. Third, the extended follow‐up periods in some studies (not defined in one study, 5.4 years in another, and between 9.9 and 13 years in the remaining five) may attenuate the measurable impact of chronic inflammation on HF risk. Fourth, some data were derived from retrospective analyses, which are inherently susceptible to residual confounding and reverse causation. Fifth, the use of crude effects may increase susceptibility to residual confounding; using adjusted effects was not possible given that statistical models and the set of confounders that were adjusted for differed across included studies. Finally, definitions for periodontitis or PD varied across the included studies; our sensitivity analyses by excluding studies using periodontitis instead of PD found that the higher risk of HF still was significant.

Our findings reinforce the potential link between PD or periodontitis and HF, highlighting a 58%–73% increased risk in individuals with PD/periodontitis compared to those without. This study extends the literature by including a larger pooled population and critically assessing bias and heterogeneity in the included studies. Although our results indicate a trend, they underscore the need for high‐quality, prospective research to confirm this association and elucidate the underlying mechanisms.

## 5. Conclusion

Our study suggests an association between PD or periodontitis, and an increased risk of HF, although high heterogeneity and a moderate‐to‐high risk of bias among the included studies were observed. These findings highlight the need for further prospective studies to validate this association and investigate its mechanistic basis. Early identification and management of PD in primary care may offer an opportunity to mitigate cardiovascular risk, though definitive evidence is required before translating this into firm clinical recommendations.

## Author Contributions

**German Valenzuela-Rodríguez**: conceptualization, data curation, formal analysis, investigation, methodology, project administration, resources, validation, visualization, writing – original draft preparation, and writing – reviewing and editing. **Carlos Diaz-Arocutipa**: data curation, formal analysis, investigation, writing – original draft preparation, software supervision, and writing – reviewing and editing. **Frank Mayta-Tovalino**: conceptualization, data curation, investigation, resources, supervision, writing – original draft preparation, and writing – reviewing and editing. **Adrian V. Hernandez**: conceptualization, data curation, investigation, resources, supervision, writing – original draft preparation, and writing – reviewing and editing.

## Acknowledgments

The authors have nothing to report.

## Funding

The study did not receive any funding.

## Disclosure

The authors have nothing to report.

## Ethics Statement

The authors have nothing to report.

## Consent

The authors have nothing to report.

## Conflicts of Interest

The authors declare no conflicts of interest.

## Supporting Information

Additional supporting information can be found online in the Supporting Information section.

## Supporting information


**Supporting Information** Search Strategies in PubMed, Embase, Scopus, and Web of Science. Figure S1: Association of periodontal disease (PD) and heart failure described with RR across all included studies. Squares represent the RR of each individual RCT, horizontal lines represent the 95% confidence intervals (CI) of the RR, and diamonds are the RR of the overall random effects meta‐analysis. Figure S2: Association of periodontitis and heart failure described with RR across all included studies. Squares represent the RR of each individual RCT, horizontal lines represent the 95% confidence intervals (CI) of the RR, and diamonds are the RR of the overall random effects meta‐analysis.

## Data Availability

The data that support the findings of the study are available from the corresponding author upon reasonable request.
