# Supplementary material for: Periodontal Disease and Risk of Heart Failure: A Systematic Review and Meta‐Analysis
Source: Int J Dent. 2026 Apr 22;2026:3288710. doi: 10.1155/ijod/3288710 (PMC13102085; doi:10.1155/ijod/3288710)

**Supplementary material**

*Search strategies*

Pubmed:

("heart failure"[mesh] OR "heart failure"[tiab] OR “heart insufficiency”[tiab] OR “cardiac failure”[tiab] OR “cardiac insufficiency”[tiab]) AND ("Periodontal Diseases"[MeSH Terms] OR periodont*[tiab] OR Parodont*[tiab] OR "pyorrhea alveolaris"[tiab] OR "Furcation Defects"[MeSH Terms] OR "Gingivitis"[MeSH Terms] OR "peri-implantitis"[MeSH Terms] OR "Periodontitis"[MeSH Terms] OR "Alveolar Bone Loss"[MeSH Terms] OR "Periodontal Attachment Loss"[MeSH Terms] OR "defect furcation"[tiab] OR "defects furcation"[tiab] OR "Furcation Defect"[tiab] OR Gingiv*[tiab] OR Periimplant*[tiab] OR Pericement*[tiab] OR "Alveolar Bone Losses"[tiab] OR "Alveolar Process Atrophy"[tiab] OR "Alveolar Process Atrophies"[tiab] OR "Alveolar Resorption"[tiab] OR "resorption alveolar"[tiab] OR "resorption periodontal"[tiab] OR "Alveolar Bone Atrophy"[tiab] OR "bone loss alveolar"[tiab])

Embase:

('heart failure' OR 'heart failure':ti,ab,kw OR hf:ti,ab,kw) AND ('periodontal disease' OR periodont*:ti,ab,kw OR parodont*:ti,ab,kw OR 'pyorrhea alveolaris':ti,ab,kw OR 'defect furcation':ti,ab,kw OR 'defects furcation':ti,ab,kw OR 'furcation defect':ti,ab,kw OR gingiv*:ti,ab,kw OR periimplant*:ti,ab,kw OR pericement*:ti,ab,kw OR 'alveolar bone losses':ti,ab,kw OR 'alveolar process atrophy':ti,ab,kw OR 'alveolar process atrophies':ti,ab,kw OR 'alveolar resorption':ti,ab,kw OR 'resorption alveolar':ti,ab,kw OR 'resorption periodontal':ti,ab,kw OR 'alveolar bone atrophy':ti,ab,kw OR 'bone loss alveolar':ti,ab,kw)

Scopus:

TITLE-ABS-KEY ("heart failure" OR HF) AND (periodont* OR Parodont* OR "pyorrhea alveolaris" OR "defect furcation" OR "defects furcation" OR "Furcation Defect" OR Gingiv* OR Periimplant* OR Pericement* OR "Alveolar Bone Losses" OR "Alveolar Process Atrophy" OR "Alveolar Process Atrophies" OR "Alveolar Resorption" OR "resorption alveolar" OR "resorption periodontal" OR "Alveolar Bone Atrophy" OR "bone loss alveolar")

Web of Science

TS=("heart failure" OR HF) AND (periodont* OR Parodont* OR "pyorrhea alveolaris" OR "defect furcation" OR "defects furcation" OR "Furcation Defect" OR Gingiv* OR Periimplant* OR Pericement* OR "Alveolar Bone Losses" OR "Alveolar Process Atrophy" OR "Alveolar Process Atrophies" OR "Alveolar Resorption" OR "resorption alveolar" OR "resorption periodontal" OR "Alveolar Bone Atrophy" OR "bone loss alveolar")

**Figure S1.** Association of periodontal disease (PD) and heart failure described with RR across all included studies. Squares represent the RR of each individual RCT, horizontal lines represent the 95% confidence intervals (CI) of the RR, and diamonds are the RR of the overall random effects meta-analysis.


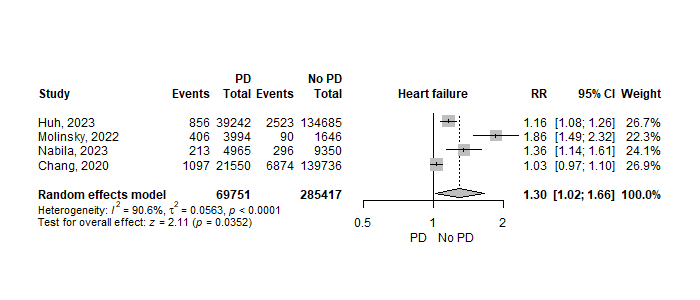


**Figure S2.** Association of periodontitis and heart failure described with RR across all included studies. Squares represent the RR of each individual RCT, horizontal lines represent the 95% confidence intervals (CI) of the RR, and diamonds are the RR of the overall random effects meta-analysis.


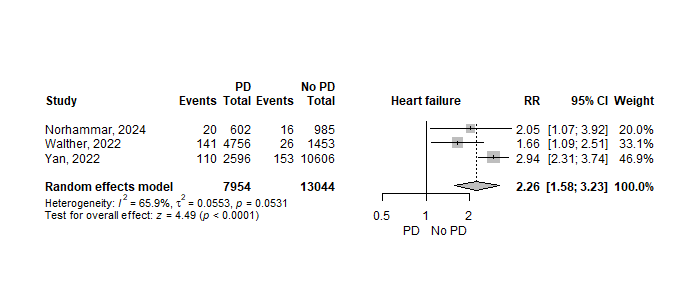

Supplement: Supplementary file 1 — Supporting Information Search Strategies in PubMed, Embase, Scopus, and Web of Science. Figure S1: Association of periodontal disease (PD) and heart failure described with RR across all included studies. Squares represent the RR of each individual RCT, horizontal lines represent the 95% confidence intervals (CI) of the RR, and diamonds are the RR of the overall random effects meta‐analysis. Figure S2: Association of periodontitis and heart failure described with RR across all included studies. Squares represent the RR of each individual RCT, horizontal lines represent the 95% confidence intervals (CI) of the RR, and diamonds are the RR of the overall random effects meta‐analysis. [file IJOD-2026-3288710-s001.docx]
